# Submuscular Plate Stabilisation After Lengthening: Standard and Modified Techniques

**DOI:** 10.5704/MOJ.2003.008

**Published:** 2020-03

**Authors:** I Munajat, AR Sulaiman, EF Mohd, MSF Zawawi

**Affiliations:** Department of Orthopaedics, Universiti Sains Malaysia, Kubang Kerian, Malaysia

**Keywords:** plating after lengthening, modified, standard

## Abstract

**Introduction:**

Submuscular plating after lengthening shortened the period of external fixation in distraction osteogenesis of the femur. In the femur, where monolateral or ring fixators had been used for the distraction, plates, could be inserted laterally, anteriorly or medially. Specific technical modification of the plate insertion, however, would be necessary to accommodate the femoral varus angular correction created at the end of the distraction, in the pelvic support osteotomy lengthening.

**Material and Methods:**

We reviewed a series of eight cases with standard and modified techniques of plating after lengthening. The amount of lengthening, the period of distraction, the external fixator index and the associated complications were assessed.

**Results:**

The mean lengthening was 5cm, with a range of 3cm to 9cm. The external fixation index, the period of external fixators in days in relation to the length of distraction in cm, was between 18 days/cm to 58 days/cm. One patient with quadriceps contracture, underwent quadriceplasty to improve knee flexion. Three patients with transient knee stiffness had resolution with aggressive physiotherapy. One patient with transient hypoesthesia recovered spontaneously. None of the patients developed joint subluxation, deep infection, re-fracture or implant failures.

**Conclusion:**

Standard and modified techniques of plating after lengthening were safe and required only a short period of external fixation. The modified technique offered an easier way of plate insertion in a deformed bone.

## Introduction

Distraction osteogenesis with an external fixator, introduced by Ilizarov, has advanced the management of limb length discrepancy^1^. Ring and monolateral fixators have been widely used as limb lengthening devices, despite their known complications^2, 3, 4^. Prolonged use of an external fixator for lengthening could result in complications of pin tract infection, pain, joint contractures, and interference with the daily and social lives of the patients^2, 3, 4^.

Limb lengthening over an intramedullary nail and submuscular plating after lengthening, could allow for early removal of the external fixators^2, 4, 5^. Both methods provided mechanical stability to support the newly formed bone structure after lengthening and possessed as well the ability to preserve periosteal blood supply^3, 5^. Lengthening over nail technique, however, could not be used in a patient with a narrow medullary canal, an open physis, a complex angular deformity or a short long bone. In these conditions, submuscular stabilisation with plating after lengthening (PAL) would be an option, and in the femur where monolateral or ring fixators had been used for distraction, the plates could be inserted laterally, anteriorly or medially.

The specific technical modification of plate stabilisation however would be necessary for a femoral varus angular correction seen at the end of the distraction of the bone lengthening, following pelvic support osteotomy. We share our experience of a submuscular PAL in eight cases using the standard technique, and a modified procedure with a pre-contouring of the plate to give an easier plate insertion in a deformed bone.

## Materials and Methods

We retrospectively reviewed seven femoral and one tibial lengthening procedures using the PAL technique in our centre. The mean age was 23.6 years (range 15-30 years). The cases included neglected developmental dislocation of the hip (DDH), non-union, physeal arrest and fibrous dysplasia (Table I). We reviewed the amount of lengthening; the waiting period which was defined as the time spent before plating once distraction was complete^1, 2^; the external fixator index (EFI), the time between the application of the external fixator and its removal, divided by the amount of lengthening in centimetres^2, 3^; the healing index, the time between the implementation of the external fixator and the time of the bony consolidation divided by the amount of lengthening in centimetres^1, 2, 3, 4^; and the associated complications.

**Table I:** Data of patients underwent lengthening with submuscular plating technique

No.	Age (years)/ Sex	Diagnosis	Technique/ Procedure	Distraction length (cm)	Distraction period (days), (days/ cm)	Waiting period (days)	PWE (day)/ EFI (days/cm)/ HI (days/cm)	Complications/ Added procedure
1	30/F	Neglected DDH	Modified technique PSO + Femoral lengthening + gradual varus creation	4	143 (36)	55	198/50/71	Transient , hypoesthesia,TKS
2	29/F	Neglected DDH	Modified technique PSO + Femoral lengthening + acute varus creation	3	30(10)	29	59/20/48	None
3	27/F	Neglected DDH	Modified technique PSO + Femoral lengthening + acute varus creation	9	256 (28)	273	529/58/78	PC/repeat corticotomy KJS/JQ
4	17/M	Fibrous dysplasia with shortening and valgus deformity of distal femur	Standard technique Femoral lengthening	5	90 (18)	24	114/23/64	TKS
5	15/M	Varus deformity of proximal tibia due to growth plate injury	Standard technique Gradual varus correction + Tibial lengthening	5	84 (17)	30	114/23/67	Early union fibula with distal migration
6	18/M	Proximal femoral growth arrest secondary to septic arthritis	Standard technique Femoral lengthening	3	53 (18)	18	71/24/67	TKS, Scar infection
7	27/M	Traumatic non-union with bone loss at the supracondylar femur	Standard technique Femoral lengthening	6	98 (16)	36	134/22/66	Knee stiffness
8	26/F	Neglected DDH	Standard technique Femoral lengthening	5	56 (11)	33	89/18/51	none

PWE - period of wearing external fixator; EFI - external fixator index; HI - healing index; DDH - developmental dysplasia of hip; PSO - pelvic support osteotomy; TKS - transient knee stiffness; PC - premature consolidation; KJS - knee joint stiffness; JQ - Judet quadriceplasty

**Fig. 1:**
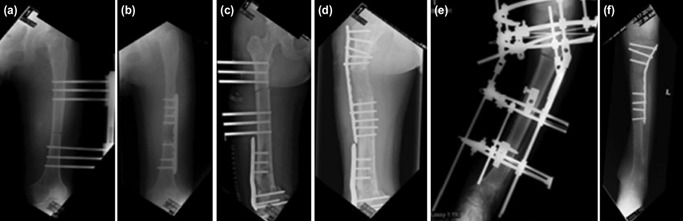
(a, b) Case no.2 that underwent standard PAL technique. (c, d) Case no.4 with angular deformity of distal femur due to fibrous dysplasia underwent corrective osteotomy of distal femur followed by lengthening with corticotomy at proximal metadiaphyseal junction and standard PAL technique. (e, f) case no.5 with proximal tibial deformity due to partial growth plate arrest underwent osteotomy at deformed area followed by standard PAL technique.

In the standard surgical technique, monolateral fixators [Orthofix-Orthofix S.R.L, Bussolengo, Verona, Italy] for femoral lengthening were placed at the anterolateral thigh to facilitate the insertion of a sub-muscular plate through a lateral plane at the end of distraction. We performed corticotomy at the distal metadiaphyseal area, and we ensured that the space in the metaphysis could accommodate three to four locking screws without injuring the physis or cartilage (Fig. 1a and 1b). In cases No. 4 and No. 7, the deformed and the non-union of the distal femur were corrected and fixed with a plate each. The corticotomy was performed at a regular segment of the proximal metadiaphyseal area (Fig. 1c and 1d). In case No. 5 with a deformity due to physeal arrest, corticotomy was done at the deformed bone; and with gradual distraction to achieve the correction of the deformity and lengthening, using Ilizarov circular rings (Fig. 1e and 1f). Corticotomy was carried out by the standard low energy technique using a 3.2mm drill bit and completed with distraction. After a latency period of 10 to 14 days, distraction osteogenesis was started at the recommended rate of one-quarter turn (0.25mm) four times a day. At the end of the distraction phase, a locking compression plate was inserted submuscular under fluoroscopy guidance as described by Uysal *et al*^6^. The plate length selected could accommodate three screws (six cortices) in each section, proximal and distal, to the distracted segment. After plating, the external fixator was then removed.

**Fig. 2:**
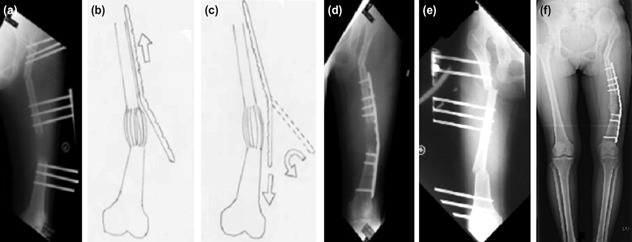
Patients with neglected DDH who went through pelvic support osteotomy. (a) The angulated femur at the end of distraction. (b, c) Schematic diagram showing pre-contoured locking plate was slid proximally and sub-muscularly with the inner surface of the plate facing outside. The plate was turned 180 degrees to correctly reposition the inner surface of the plate onto the bone, followed by advancing it more distally. (d) The final position of the plate after fixation. (e) Post-op radiograph of another patient after pelvic support osteotomy, the angle of proximal valgus should be equal to the angle of maximum adduction plus overcorrection in the region of 25-30 degrees to allow a shift of the limb from the midline. Compensatory distal varus is created to realign the femoral shaft or knee sufficiently in the weight-bearing axis. (f) Post-op standing radiograph of case no. 1 with acceptable alignment.

An innovative modified surgical technique was used in three patients who underwent pelvic support osteotomy together with the lengthening of the femur. The monolateral fixator pins were adjusted and placed at the true lateral site of the thigh to allow for the creation of a varus to correct the mechanical axis (Fig. 2a and 2e). Upon achieving the desired length, the pins were repositioned to the anterolateral site and left for a period, to allow the healing of the lateral pin tract before the insertion of the submuscular plate. The technique of the submuscular plating in these patients was modified by inserting a pre-contoured locking plate at the apex of angulation and sliding proximally with the inner surface of the plate facing outside (Fig. 2b and 2c). The plate was then flipped 180° to correctly reposition the inner surface of the plate onto the bone followed by advancing the plate distally across the distracted part before inserting the screws (Fig. 2c and 2d).

Post-operatively, the patients received physiotherapy to the knee and the ankle to prevent joint stiffness as well as to maintain muscle strength. Patients were allowed partial weight-bearing ambulation as tolerated. Full weight-bearing was allowed in both techniques once the lengthened and the osteotomy sites of the bone showed solid consolidation on plain radiograph.

## Results

The mean lengthening was 5cm (3 to 9cm). The mean distraction period was 19 days/cm (10 to 36 days/cm). The distraction period for case No. 1 was 36 days/cm, which was prolonged due to a technical problem during gradual varus creation. The mean waiting period was 57 days (18 to 273 days). The waiting period for case No.3 took 273 days which was longer than the other cases because PAL had been unplanned, and the patient required Judet quadriceplasty for the knee. The mean EFI was 30 days/cm (18 to 58 days/cm).

Three patients had a superficial pin-track infection that resolved with wound care and antibiotics. There was no clinical and radiological evidence of infection around the plate throughout the consolidation phase.

In the standard technique, there was no proximal migration of the dislocated femoral head in case No. 8 with a neglected DDH after femoral lengthening without fixation across the hip joint. One patient, Case No. 6, had a superficial infection over the surgical scar at the end of the consolidation phase after PAL. The plate was removed at the end of the consolidation phase, and there was no evidence of deep infection in the soft tissue and bone intra-operatively. Distal migration of the proximal fibula due to an early union of the fibula osteotomy was observed in case No. 5 during lengthening of the ipsilateral tibia, with no common peroneal nerve palsy. In this case, we used a ring fixator to correct the angular deformity of the tibia before lengthening (Fig. 1e and 1f). Two patients, cases No. 4 and 6, had transient knee stiffness that improved with aggressive physiotherapy.

With the modified technique, one patient, case No. 3, had limited knee flexion, from 0° to 30°. This patient underwent Judet quadriceplasty followed by physiotherapy and finally gained a limited range of knee movement. There was a premature consolidation resulting in residual shortening of more than 2.5cm in case No. 3. Corticotomy was repeated, followed by re-distraction to achieve the desired length. Temporary hypoesthesia occurred in one patient, case No. 1, that resolved spontaneously at the end of the distraction phase. Compared to the standard technique, only one patient, case No. 1 had transient knee stiffness that improved with aggressive physiotherapy in the modified technique. There was no pain around the hip and the groin in all the three patients that would suggest an abutment of the proximal part of the femur against the pelvis. All of them had improvement in the Trendelenburg gait.

Once angular deformity and shortening of the tibia were corrected, PAL was carried out. All patients were satisfied with standard or near-normal equalisation of the limbs with acceptable function during their latest follow-ups.

## Discussion

The key differences between the standard PAL technique and the modified technique were the placement of the pins and the insertion of a plate. In the standard procedure, the external fixator pins were placed in the anterolateral thigh to allow insertion of a submuscular plate at the end of the distraction. In the modified technique, the external fixator pins were placed in a true lateral plane. The laterally placed pins allowed better knee rehabilitation during lengthening, as the pins pierced a smaller bulk of quadriceps laterally, compared to the anterolaterally placed pins which tethered a greater bulk of the quadriceps, preventing a free gliding movement. However, additional surgery was required to convert the external fixator pins to the anterolateral plane and additional time for wound healing before the submuscular plate could be inserted. A true lateral plane placement of the pins was also critical in the pelvic support osteotomy since only laterally placed pins created a true varus angular correction without any component of flexion or extension at the distraction site. The varus angular correction of the distal femur was needed to compensate for the extra valgus of the proximal femur, in cases No. 1, 2 and 3

The standard technique of submuscular plate insertion had been very well described for normal bone anatomy through a distal or a proximal surgical wound^6, 7^. This technique was used in all cases and resulted in an anatomical bony shape at the end of distraction. In cases with a varus correction at the end of distraction, as in cases No. 1, 2 and 3, we modified the technique by inserting the varus pre-bend plates from the lateral wound at the apex of the deformity. This approach allowed the plate to be slid submuscular proximally and then flipped back and slid distally without disturbing the newly formed bone. This technique made the plate insertion and stabilisation much easier. There were no specific pitfalls and complications related to the modified technique.

If the plate was inserted using the standard technique, in cases No. 1-3, this unbent plate would not accommodate with the contour of the distal varus created earlier; it would be difficult to fix and stabilise the newly regenerated bone. However, the prebent plate with the modified technique could slide easily following the varus contour of the distal femur, and the fixation would be hassle-free.

The use of medial plating^8^ and anterior plating technique in distraction osteogenesis of the femur^9^ had been reported to avoid previous pin sites to minimise the risk of infection. Still, the medial plate and anterior plate fixation would not be suitable for the deformed varus bone in pelvic support osteotomy lengthening in cases No. 1, 2 and 3. In case No. 4 with valgus deformity secondary to the fibrous dysplasia, corrective osteotomy of the supracondylar femur followed by fixation with a retrograde intramedullary femoral nail and distraction over nail could be an option. However, we tried to avoid distraction osteogenesis over fibrous dysplasia lesion at the distal femur due to doubtful bone regeneration. In the non-union supracondylar fracture in case No. 7, the pre-existing knee stiffness with knee flexion range of less than 20° prevented the use of a retrograde femoral nail. Therefore, both patients underwent corrective osteotomy over a centre of the rotational axis and open reduction with bone graft. Corticotomy was performed over the normal proximal metadiaphyseal for distraction osteogenesis followed by submuscular plating (Fig. 1c and 1d). Case No. 8 with hip dislocation with relatively stiff hips did not require pelvic support osteotomy. We noted no migration of the femoral head or knee subluxation in these cases despite 5cm distraction.

Performing a proximal femoral valgus osteotomy equal in size to the maximum range of adduction with the distal femoral fragment aligned in weight-bearing axis without compensatory distal varus correction did not lateralise the femoral shaft or knee joint sufficiently^10^. Therefore, a proximal valgus overcorrection in the region of 30° would allow a shift of the limb from the midline, which is a valgus correction^10^.

The second osteotomy over the distal part of the femur allowed the creation of varus, thus realigning the mechanical axis of the lower limb. In other words, the second osteotomy removed the ’abduction contracture’. It allowed both limbs to be parallel, with the knee, ankle and the pelvis horizontal^10^ to avoid stretching the knee collateral ligaments. The treated side remained in maximum adduction at its articulation with the pelvis and therefore prevented a Trendelenburg gait^10^. Lengthening at the second osteotomy removed limb length inequality.

The PAL technique was chosen as we preferred to lengthen the bone following the mechanical axis of the femur, which finally created a deformed femur. One of the advantages of PAL was early rehabilitation allowing a better range of joint motion. In our series, all cases regained full range of motion except case No. 7 who had pre-existing knee stiffness before lengthening.

Infection was one of the main worries when dealing with implant insertion following external fixation with a report of 10.8% of deep infection, including late osteomyelitis after a consolidation period had been completed^11^. The use of the locking plate during external fixator application and the insertion of the retaining screws on the plate during the removal of the external fixator with completion of distraction was not associated with the deep soft tissue infection and osteomyelitis^12^. No deep infection in the plating area was found with the sequential use of the internal fixator after the removal of the external fixator^6, 13^. Consistent with this, the insertion of the plate after distraction in our series was not associated with any deep infection.

Fracture after PAL technique occurred at the plate- bone-junction, which was the site of stress riser^7^. Fracture of the tibial distraction callus and failure of the locking plate attributed to less stable fixation were also reported^12^. Knowing these facts, we ensured our plates were stabilised by at least six cortices on each side and found neither fracture nor implant failure in our series. We preferred using a locking plate to ensure that an adequate number of screws could be placed in the limited space.

PAL technique was also suitable in deformed or short long bones. It had also been used to correct the deformity of the lower extremity in children to overcome the limitation of using an intramedullary nail in the narrow medullary canal and the risk of avascular necrosis of femoral head with the piriformis entrance^14^. In distraction osteogenesis without internal fixation, an external fixator would be used during the distraction and consolidation period. Catagni *et al*^15^ reported that an EFI for adult patients was 40.7 days/cm. The EFI in the PAL technique were between 20 days/cm6 and 1.3 months/cm^13^. In our case series, EFI was 18 days/cm to 58 days/cm.

The waiting period for patients in our series was between 18 to 273 days. The range of the waiting period differed because we waited for the healing of the pin tract before inserting the plate. During lengthening, the pin tracts were usually unhealthy due to skin necrosis and healing times varied with the different grades of infection, where Grade III and Grade II pin tract infections significantly delayed plate insertion^6^. Endo *et al*^16^ suggested postponing the conversion from external fixation to internal fixation if blood test and clinical findings indicated an active infection.

Milch *et al*^17^ and Adams *et al*^18^, advised against excessive valgus osteotomy at pelvic support osteotomy, as excessive proximal valgus osteotomy could limit the range of adduction and lead to a valgus malalignment of the knee. Ilizarov provided a solution with a second more distal femoral osteotomy^19, 20^. This second osteotomy realigned the knee joint with a varus angular correction simultaneously equalising the lower extremity length discrepancy^19, 20^.

Ilizarov^19^, also stated that in pelvic support osteotomy, the mean loss of correction at the osteotomy site was 3° to 13° for patients between 9 and 17 years of age. Similarly, Rozbruch *et al*^21^, noted that when pelvic support osteotomy was performed on a young patient, remodelling of the proximal osteotomy site should be expected, and the procedure would need to be repeated. In all our pelvic support osteotomy cases, even though none were children, extra valgus on the proximal osteotomy provided better hip stability and avoided the medialising of the entire limb closer to the midline than the contralateral normal side.

## Conclusion

The standard and the modified techniques of submuscular plating after lengthening were safe with a shorter fixator-free period. The modified technique offered an easier way of plate insertion in a deformed bone. Both allowed early limb rehabilitation and protected the regenerated bone throughout the consolidation phase.

## References

[1] Ilizarov GA (1990). Clinical application of the tension-stress effect for limb lengthening.. Clin Orthop Relat Res..

[2] Paley D (1990). Problems, obstacles, and complications of limb lengthening by the Ilizarov technique.. Clin Orthop Relat Res..

[3] Paley D, Herzenberg JE, Paremain G, Bhave A (1997). Femoral lengthening over an intramedullary nail. A matched-case comparison with Ilizarov femoral lengthening.. J Bone Joint Surg Am..

[4] Dahl MT, Gulli B, Berg T (1994). Complications of limb lengthening. A learning curve.. Clin Orthop Relat Res..

[5] Oh CW, Shetty GM, Song HR, Kyung HS, Oh JK, Min WK (2008). Submuscular plating after distraction osteogenesis in children.. J Pediatr Orthop B.

[6] Uysal M, Akpinar S, Cesur N, Hersekli MA, Tandoğan RN (2007). Plating after lengthening (PAL): technical notes and preliminary clinical experiences.. Arch Orthop Trauma Surg..

[7] Iobst CA, Dahl MT (2007). Limb lengthening with submuscular plate stabilisation: a case series and description of the technique.. J Pediatr Orthop..

[8] Nayagam S, Davis B, Thevendran G, Roche AJ (2014). Medial submuscular plating of the femur in a series of paediatric patients: a useful alternative to standard lateral techniques.. Bone Joint J..

[9] Persico F, Fletscher G, Zuluaga M (2017). Submuscular plating of the femur through an anterior approach after bone distraction.. Strategies Trauma Limb Reconstr..

[10] Pafilas D, Nayagam S (2008). The pelvic support osteotomy: indications and preoperative planning.. Strategies Trauma Limb Reconstr..

[11] Gordon JE, Manske MC, Lewis TR, O’Donnell JC, Schoenecker PL, Keeler KA (2013). Femoral lengthening over a pediatric femoral nail: results and complications.. J Pediatr Orthop..

[12] Oh CW, Song HR, Kim JW, Choi JW, Min WK, Park BC (2009). Limb lengthening with a submuscular locking plate.. J Bone Joint Surg Br..

[13] Harbacheuski R, Fragomen AT, Rozbruch SR (2012). Does lengthening and then plating (LAP) shorten duration of external fixation?.. Clin Orthop Relat Res..

[14] Buford D Jr, Christensen K, Weatherall P (1998). Intramedullary nailing of femoral fractures in adolescents.. Clin Orthop Relat Res..

[15] Catagni MA, Lovisetti L, Guerreschi F, Combi A, Ottaviani G (2005). Cosmetic bilateral leg lengthening: experience of 54 cases.. J Bone Joint Surg Br..

[16] Endo H, Asaumi K, Mitani S, Noda T, Minagawa H, Tetsunaga T (2008). The minimally invasive plate osteosynthesis (MIPO) technique with a locking compression plate for femoral lengthening.. Acta Med Okayama.

[17] Milch H (1989). The “pelvic support” osteotomy. 1491.. Clin Orthop Relat Res..

[18] Adams JC (1985). Standard orthopaedic operations.

[19] Ilizarov GA (1992). Transosseous Osteosynthesis.

[20] Ilizarov GA, Samchukov ML (1988). Reconstruction of the femur by the Ilizarov method in the treatment of arthtosis deformans of the hip joint.. Ortop Travmatol Protez..

[21] Rozbruch SR, Paley D, Bhave A, Herzenberg JE (2005). Ilizarov hip reconstruction for the late sequelae of infantile hip.. J Bone Joint Surg Am..

